# Author Correction: Caveolin-1 promotes invasion and metastasis by upregulating Pofut1 expression in mouse hepatocellular carcinoma

**DOI:** 10.1038/s41419-019-1800-1

**Published:** 2019-07-29

**Authors:** Cheng Zhang, Huang Huang, Junshi Zhang, Qiong Wu, Xixi Chen, Tianmiao Huang, Wenli Li, Yubo Liu, Jianing Zhang

**Affiliations:** 1 School of Life Science & Medicine, Dalian University of Technology, Panjin, China; 2 School of Life Science & Biotechnology, Dalian University of Technology, Dalian, China

**Keywords:** Glycobiology, Metastasis, Cell signalling

**Correction to: Cell Death Disease**

10.1038/s41419-019-1703-1 published online 17 June 2019

Since publication of this paper, the authors have noticed an error in one of the images. In Fig. 2c, the graphs of c-Myc and CREB are incorrect. During image synthesis, these two images were confused with other images from the raw data. The error does not impact the conclusions of the article. The authors would like to apologize for any inconvenience this may have caused.

**Fig. 2:**
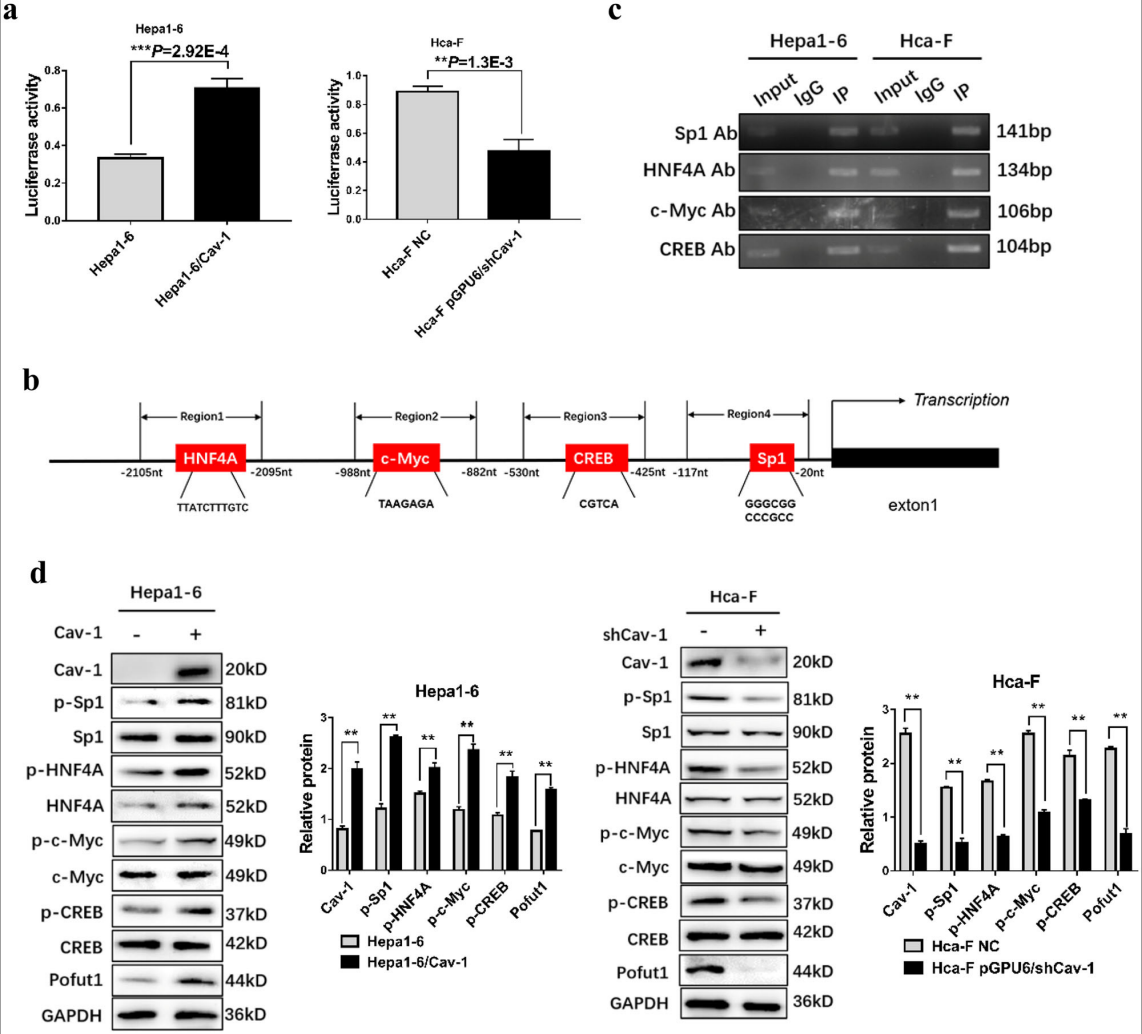
∎

The correct figures are presented here.

This has been corrected in both the PDF and HTML versions of the Article.

